# Randomised trial of a parent‐mediated intervention for infants at high risk for autism: longitudinal outcomes to age 3 years

**DOI:** 10.1111/jcpp.12728

**Published:** 2017-04-10

**Authors:** Jonathan Green, Andrew Pickles, Greg Pasco, Rachael Bedford, Ming Wai Wan, Mayada Elsabbagh, Vicky Slonims, Teea Gliga, Emily Jones, Celeste Cheung, Tony Charman, Mark Johnson, The British Autism Study of Infant Siblings (BASIS) Team, Simon Baron‐Cohen, Patrick Bolton, Kim Davies, Michelle Liew, Janice Fernandes, Isobel Gammer, Erica Salomone, Helena Ribeiro, Leslie Tucker, Carol Taylor, Rhonda Booth, Claire Harrop, Samina Holsgrove, Janet McNally

**Affiliations:** ^1^ Social Development Research Group School of Biological Sciences University of Manchester Manchester UK; ^2^ Royal Manchester Children's Hospital Manchester UK; ^3^ Institute of Psychiatry, Psychology and Neuroscience King's College London London UK; ^4^ National Institute for Health Research Medical Health Biomedical Research Centre South London and Maudsley NHS Foundation Trust London UK; ^5^ Centre for Brain and Cognitive Development Birkbeck College London UK; ^6^ School of Health Sciences University of Manchester Manchester UK; ^7^ Department of Psychiatry McGill University West Montréal QC Canada; ^8^ Evelina London Children's Hospital and King's College London Neurosciences Centre London UK

**Keywords:** Pre‐emptive intervention, prevention trials, autism, autism spectrum disorder, high‐risk siblings, parent‐mediated intervention

## Abstract

**Background:**

There has been increasing interest in the potential for pre‐emptive interventions in the prodrome of autism, but little investigation as to their effect.

**Methods:**

A two‐site, two‐arm assessor‐blinded randomised controlled trial (RCT) of a 12‐session parent‐mediated social communication intervention delivered between 9 and 14 months of age (Intervention in the British Autism Study of Infant Siblings‐Video Interaction for Promoting Positive Parenting), against no intervention. Fifty‐four infants (28 intervention, 26 nonintervention) at familial risk of autism but not otherwise selected for developmental atypicality were assessed at 9‐month baseline, 15‐month treatment endpoint, and 27‐ and 39‐month follow‐up. Primary outcome: severity of autism prodromal symptoms, blind‐rated on Autism Observation Schedule for Infants or Autism Diagnostic Observation Schedule 2nd Edition across the four assessment points. Secondary outcomes: blind‐rated parent–child interaction and child language; nonblind parent‐rated communication and socialisation. Prespecified intention‐to‐treat analysis combined estimates from repeated measures within correlated regressions to estimate the overall effect of the infancy intervention over time.

**Results:**

Effect estimates in favour of intervention on autism prodromal symptoms, maximal at 27 months, had confidence intervals (CIs) at each separate time point including the null, but showed a significant overall effect over the course of the intervention and follow‐up period (effect size [ES] = 0.32; 95% CI 0.04, 0.60; *p* = .026). Effects on proximal intervention targets of parent nondirectiveness/synchrony (ES = 0.33; CI 0.04, 0.63; *p* = .013) and child attentiveness/communication initiation (ES = 0.36; 95% CI 0.04, 0.68; *p* = .015) showed similar results. There was no effect on categorical diagnostic outcome or formal language measures.

**Conclusions:**

Follow‐up to 3 years of the first RCT of a very early social communication intervention for infants at familial risk of developing autism has shown a treatment effect, extending 24 months after intervention end, to reduce the overall severity of autism prodromal symptoms and enhance parent–child dyadic social communication over this period. We highlight the value of extended follow‐up and repeat assessment for early intervention trials.

## Introduction

### Pre‐emptive intervention in developmental disorder

A decade of prospective studies of infants at familial risk of developing autism in the first 3 years of life has made substantial advances towards a developmental account of the emergence of autism, with deeper understanding of the phenotype at brain as well as behavioural level (Johnson, Gliga, Jones, & Charman, 2015; Szatmari et al., 2016). These advances, in turn, suggest possibilities for intervention in the autism prodrome, targeting either the earliest behavioural symptoms of emerging disorder or those neurocognitive biomarkers that have predictive salience in early development (Dawson, 2008). There is an appealing, but unproven, argument that very early intervention in these emergent trajectories may be especially effective, with added potential to exploit early brain system plasticity to altered environmental input (Johnson, 2011).

Intervention of this kind can be seen in a wider context of ‘prevention’ studies in mental health and biomedicine, in which risk biomarkers are identified as targets for a range of intervention strategies: universal*,* targeted at a whole population group; selective, at individuals at high risk (HR) of a condition, or indicated*,* at those showing early symptoms indicating predisposition but without meeting diagnostic criteria (O'Connell, Boat, & Warner, 2009). Autism prevalence rates preclude the feasibility of ‘universal’ strategies, and selective or indicated interventions are the basis of current work. But, furthermore, the very aim of simple ‘prevention’ is not now considered realistic or appropriate. Insel (2007) coined the alternative term ‘pre‐emptive intervention’, suggesting a strategy to mitigate developmental risks and modify prodromal symptom trajectories, rather than ‘eliminate’ a condition. We use this latter term as reflecting the intent of our work. In pre‐emptive intervention, the combination of a randomised controlled trial (RCT) of a theoretically targeted intervention with repeated measures follow‐up can also be a powerful method of illuminating causal processes in developmental science, and testing predictive models alongside clinical effectiveness (Howe, Reiss, & Yuh, 2002).

Apart from the Intervention in the British Autism Study of Infant Siblings (iBASIS) study discussed in this paper, the extant literature on early pre‐emptive autism spectrum disorder (ASD) studies in the first year of infancy consists wholly of case‐series (*n *=* *3–8; Bradshaw, Steiner, Gengoux, & Koegel, 2015). Some are ‘selective interventions’ with infants at known familial risk of autism (Green et al., 2013; Steiner, Gengoux, Klin, & Chawarska, 2013), others are ‘indicated interventions’ with infants assessed as having emergent atypicalities thought by the investigators to be autism‐related (although empirical evidence of this is limited); for instance ‘early signs of difficulty in relation to affect, social interest, eye contact avoidance or response’ (Koegel, Singh, Koegel, Hollingsworth, & Bradshaw, 2013) or ‘risk indicators’ including unusual patterns of attention, repetitive behaviours or eye contact (Rogers et al., 2014). In the second year, there are RCT studies that do still qualify as indicated pre‐emptive studies, as they sample toddlers screening positive for autism‐related atypicality prior to diagnosis; all used forms of parent‐focussed intervention, with mixed results. A small study at mean age 15 months (*n *=* *16, Baranek et al., 2015) reported endpoint findings on infant receptive language (RL) and parent‐reported socialisation, but no long‐term effects. A 3‐month ‘Hanen’ group parent training at mean 20 months (*n* = 62; Carter et al., 2011) produced near‐significant effect on parental interaction but no child effects, although low baseline object interest moderated better child outcome. A 3‐month therapist–parent coaching model at mean 21 months (Early Start Denver Model for parents, *n* = 98; Rogers et al., 2012) had no effect on parent or child variables despite increased parent–therapist engagement; and a 3‐month ‘focussed playtime intervention’ at mean 22 months (*n* = 66; Kasari et al., 2014) showed improved parent responsiveness but no effect on child joint attention or social interaction. Jones, Dawson, Kelly, Estes, and Webb (2017) have recently published an infancy RCT (*n* = 33) of a parent intervention with evidence of some positive effects on infant neurophysiological markers.

### Intervention within the British Autism Study of Infant Siblings

The iBASIS trial was designed as a selective pre‐emptive trial; including infants at high familial autism‐risk due to having a sibling with the condition, but not otherwise selected on the basis of developmental atypicality. The timing of the intervention was set between 9 and 14 months when results from previous studies suggest that early atypicalities at the brain and cognitive level first emerge (Johnson et al., 2015; Szatmari et al., 2016), when the parent–child interaction context is central to infant social development (Tomasello, 2008), and there is evidence of alterations in parent–child interaction related to at‐risk status. An independent BASIS cohort study showed reduced parental ‘responsiveness’ and ‘nondirectiveness’ with 7‐month at‐risk infants compared to low‐risk controls (Wan et al., 2012); at 14 months, these differences continued, but were accompanied at that point by decreased infant attentive engagement to parent, affect sharing and mutuality; and these infant interactive behaviours then predicted ASD emergence at 3 years (Wan et al., 2013). Evidence that the 7‐month effects are associated with altered neurophysiological visual social processing in the infant (Elsabbagh et al., 2014) and that infant rather than parent interactional qualities predict later ASD emergence, support the idea that these observed interactional cycles are initially evoked by an atypical infant development, but could then feedback to alter the infant's further social learning, thus amplifying pre‐existing vulnerability. This does not imply primary parenting difficulties, but rather that contingent responses are more challenging for parents in the context of infant with atypical development (Slonims, Cox, & McConachie, 2006). In clinical terms, parent–infant interaction that is attuned and in which the parent is able to ‘read’ the child's communicative signals promotes positive social and communicative development in all children. Infants at‐risk for autism often show ‘weak’ or distorted communicative signals which parents can struggle to recognise and respond to accurately. The iBASIS intervention was designed specifically to reverse such disrupted patterns of early parent–infant interaction, with the hypothesis that there would be consequent positive effects on other infant developmental markers and emerging prodromal autism symptoms.

Building on an initial case‐series feasibility study (Green et al., 2013), we tested the efficacy of the time‐limited (5 month), parent‐mediated intervention for 7‐ to 10‐month‐old infants (mean 9 months) at familial high risk of autism in a two‐site, two‐arm RCT of iBASIS against usual care (*n* = 54). The 15‐month treatment endpoint results (Green et al., 2015) showed wide effect size (ES) confidence intervals (CIs) along with the modest sample size. There was significant increase in parent nondirectiveness (ES = 0.81), the proximal target of the parent‐mediated intervention, but less clear impact on child measures of child attentiveness, communication initiation and autism‐related behaviours on the Autism Observation Scale for Infants (AOSI; ES = 0.50), which all showed positive point estimates of effect but with CIs crossing the null. There was an unexpected nonsignificant trend towards negative effect on a range of receptive, expressive language (EL) and communication scores.

### The current study

The current manuscript extends these previously reported findings in two principal ways. First, we report, for the first time in the literature, data on subsequent planned follow‐up to age 27 and 39 months, including prodromal autism‐related behaviours and clinical categorical diagnostic outcome. Second, we report a longitudinal repeated measures analysis that not only highlights the time‐paths of the effects of intervention, giving insight into the possible therapeutic and developmental mechanisms but also increases the statistical power to sharpen effect estimates.

## Method

### Design

A two‐site, single (rater)‐blinded RCT of two parallel groups: intervention and no intervention. Research assessments were made at the Centre for Brain and Cognitive Development, Birkbeck College, at pre‐randomisation baseline (9 months), following 5 months of intervention (15 months; Green et al., 2015), and at 27‐ and 39‐month follow‐up. The London Research Ethics Committee approved the study (Ref: 09/H0718/14) and parents provided written informed consent. This study is registered as ISRCTN 87373263 (http://www.isrctn.com/); the trial protocol is available at http://www.bbmh.manchester.ac.uk/ibasis/protocol/.

### Allocation and masking

Details of the conduct of the trial and intervention are reported in Green et al. (2015) and in the Supporting Information. All assessments were administered and coded blind to other information including group allocation, with the exception of parent‐rated measures of adaptive behaviour.

### Participants

Siblings of autism probands were sampled within the context of a prospective longitudinal observational study, the BASIS (http://www.basisnetwork.org/) and age 7–10 months at baseline. Exclusion criteria were significant medical conditions in infant, twinship, prematurity <34 weeks or birth weight <5 lbs. Families were approached in order of identification, and infants were not selected on the basis of developmental characteristics or atypicality. Further details on participant characterisation are given in the Supporting Information.

### Intervention

The intervention was iBASIS‐Video Interaction for Promoting Positive Parenting (iBASIS‐VIPP), a modification for the autism prodrome of the VIPP infancy programme (Juffer, Bakermans‐Kranenburg, & Van IJzendoorn, 2008). The comparator group had no planned intervention. iBASIS‐VIPP uses video‐feedback to help parents understand and adapt to their infant's individual communication style to promote optimal social and communicative development (Green et al., 2015 and Supporting Information). Given the potential developmental complexity of prodromal ASD, we adapted the original six‐session infancy VIPP programme and added up to six further planned booster sessions according to need, plus procedures to address any emerging developmental atypicality. The therapist uses video excerpts of parent–child interaction in a series of developmentally sequenced home‐sessions focussing on: interpreting the infant's behaviour and recognising their intentions, enhancing sensitive responding, emotional attunement and patterns of verbal and nonverbal interaction. The intervention was carried out in participant homes in Manchester and London regions.

### Measures

#### Primary outcome – autism prodromal symptoms

Across different time points, we used two conceptually analogous measures of autism prodromal symptoms. The behaviours measured are conceptually continuous with ASD symptoms after clinical diagnosis and we refer to them therefore as ‘prodromal symptoms’, without suggesting that all children showing them develop ASD.

The AOSI (Bryson, Zwaigenbaum, McDermott, Rombough, & Brian, 2008), completed at 9‐month baseline and 15‐month endpoint, is a semistructured observational assessment of early autism‐related behaviours that are risk markers for ASD covering social reciprocity and imitation, and motor, attention and sensory behaviours. AOSI total score in 14‐ to 18‐month‐old infants is associated with later Autism Diagnostic Observation Schedule (ADOS) scores and with ASD diagnosis at 3 years (Brian et al., 2008; Gammer et al., 2015).

Autism Diagnostic Observation Schedule 2nd Edition (Lord et al., 2012), completed at the 27‐ and 39‐month assessments, is a structured assessment of experimenter–child interaction, which is used internationally as part of autism diagnostic assessment. We report ADOS‐2 combined social affect and rigid and repetitive behaviours total score as a continuous measure of emerging ASD symptomatology. Two trained raters blind to treatment allocation (one administrator, one observer) coded each observation and agreed a consensus code following the assessment.

### Secondary outcomes

#### Parent–child social interaction

Two different conceptually related coding schemes were applied over the four time points of the trial.

Manchester Assessment of Caregiver–Infant Child interaction (MACI; Wan, Brooks, Green, Abel, & Elmadih, 2016; Wan et al., 2013), is a global coding on 7‐point scales from 6‐min videotaped free‐play interaction. The infancy version of the instrument was used at the 9‐ and 15‐month assessment points and the toddler version at 27 months. MACI was designed to investigate the interactional antecedents of social competency in infancy and early parent–infant atypicalities in the autism prodrome. Based on previous work, two MACI scales, caregiver ‘nondirectiveness’ and child ‘attentiveness to caregiver’ were prespecified outcomes (for details see Supporting Information). Within‐study independent coding (*n *=* *11) showed intraclass correlations (ICC; single measures, absolute agreement) of .75 for nondirectiveness and .84 for child attentiveness.

The Dyadic Communication Measure for Autism (DCMA; Aldred, Green, Emsley, & McConachie, 2012), at 27‐ and 39‐month follow‐up, is an event coding of parent–child communication based on an 8‐min sample of parent–child free play, developed for preschool children diagnosed with autism. Two DCMA scales were prespecified for outcome as the closest analogues conceptually to MACI ‘caregiver nondirectiveness’ and ‘infant attentiveness’: ‘parental synchrony’ (the proportion of parental communications that are contingently responsive to the child) and ‘child initiations’ (the proportion of child communications with the parent that are spontaneous communication acts directed towards the parent, including nonverbal indication and verbalisation/vocalisation). Both these scales have shown sensitivity to intervention effects in trials of parent‐mediated intervention for autism in children from 2 years (Green et al., 2010; Rahman et al., 2016), and mediation effects on ADOS symptom change (Aldred et al., 2012; Pickles et al., 2015). Thirty‐three (33%) of all videos across both time points were independently double‐coded, with ICC (one‐way random, single measures) .81 (*p* < .001) for parent synchrony and .74 (*p* < .001) for child initiations. (For further details of DCMA codings and the conceptual continuities between MACI and DCMA coding constructs at each time point, see Supporting Information.)

#### Developmental measures

The Mullen Scales of Early Learning (MSEL; Mullen, 1995) are a standardised developmental assessment, examining early motor, language and cognitive development, completed at 9, 15, 27 and 39 months. RL, EL *T*‐scores were analysed.

Vineland Adaptive Behavior Scales – 2nd Edition (Sparrow, Cicchetti, & Balla, 2005), is a parent‐report measure of adaptive behaviour yielding age‐normed standard scores on communication and socialisation domains.

#### Clinical diagnosis

At the 39‐month time point, experienced researchers (TC, GP, CC) reviewed 27‐ and 39‐month data on ASD symptomatology (ADOS‐2; Autism Diagnostic Interview‐Revised; ADI‐R; Lord, Rutter, & Le Couteur, 1994), Social Communication Questionnaire (Rutter, Bailey, & Lord, 2003), adaptive functioning (Vineland) and development (MSEL) for each child, to ascertain diagnostic outcomes of: (i) ‘ASD’ using clinical best estimate consistent with DSM‐5 criteria; (ii) ‘Atypical’ development through showing (a) ADOS autism criteria alone, with or without ADI‐R (Risi et al., 2006); (b) greater than 1.5 *SD* below the population mean on the Mullen ELC (<77.5) or on the Mullen EL or RL subscales (<35; *n *=* *5); or (c) criteria (a) plus (b); (iii) the remaining participants were considered ‘typically developing’. (See Supporting Information for further details).

### Statistical analysis

The intention‐to‐treat analysis followed a statistical analysis plan for the follow‐up analysis, prespecified in outline at the design stage (see trial protocol) and in final detail after the initial 15‐month data analysis but before unblinding the 27‐ and 39‐month data. The results presented follow the analysis plan for the 39‐month follow‐up (additional results related just to the 27‐month analysis are also included in the Supporting Information). A late revision was the replacement of ADOS‐2 comparative severity score by a log‐transformed ADOS‐2 total score analysed by regression, in order to allow a uniform ES estimator (Cohen's *d*) to be used across AOSI and ADOS in the longitudinal analysis (see Results and Figure 2).

All analyses were undertaken in Stata 14 (StataCorp, 2015). Combined analysis of the treatment effect estimates available at trial baseline, 15, 27 and 39 months used seemingly unrelated regressions (Zellner, 1962) estimated by maximum likelihood using the *sem* procedure so as to include data from all 54 participants, including those with incomplete records. This method allows a set of treatment effect regressions to have different error variances and different predictors but nonetheless recognises that the regressions involve the same participants and adjusts for their correlation. Combining occasion‐specific estimates of treatment effect can give increased power, not least because it improves the reliability of the posttreatment characterisation. A Cohen's *d* ES for each measure was calculated using the within‐group standard deviation of the outcome at each assessment occasion (also within module for the 39‐month ADOS‐2). Each analysis covaried for relevant baseline measure value, plus key prespecified variables of centre, age‐at‐assessment, mother's ethnicity and educational qualifications (imbalanced at follow‐up), and treatment group assignment. To summarise the treatment group differences in a principled fashion, the multiple point estimates were combined into an area between the curves (sum of trapeziums, see Figures 2 and 3). A Wald test for this estimated area was calculated from the individual effect estimates and their parameter covariance using the lincom procedure. CIs for area ESs were obtained by bootstrap, resampling participants with replacement. Further details of the analytic method are included in the Supporting Information.

## Results

**Figure 1:**
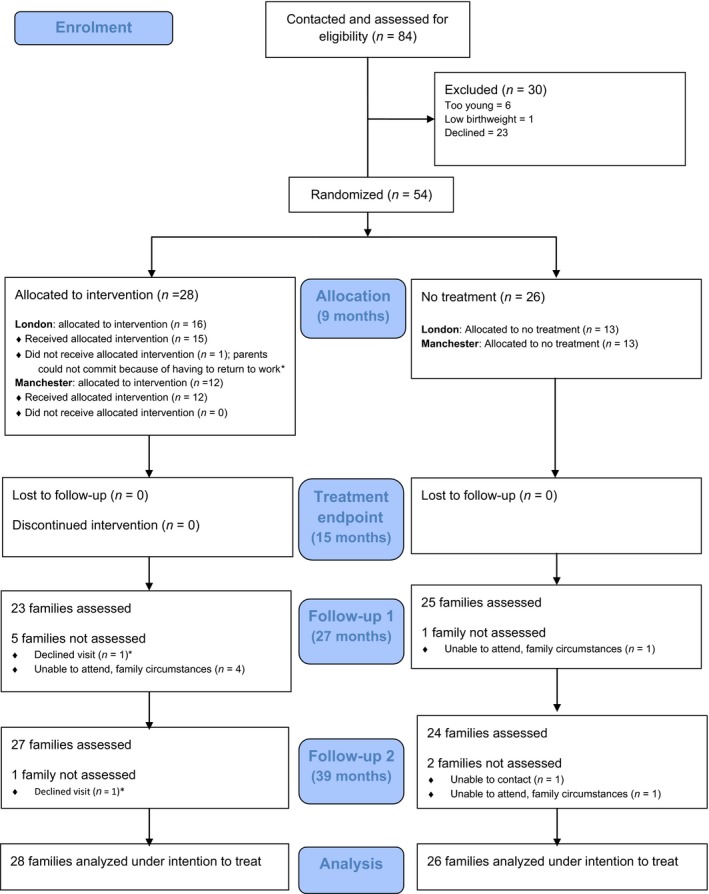
Intervention in the British Autism Study of Infant Siblings CONSORT participant flow diagram. *The same family [Colour figure can be viewed at wileyonlinelibrary.com]

Figure 1 shows the participant flow through the whole study and Table 1 shows the summary statistics for each assessment time point. Tables S1 and S2 show baseline sample descriptive characteristics and correlations across time points for each of the seven sets of measures, respectively.

**Table 1:** Baseline, 15‐month, 27‐month and 39‐month follow‐up data by intervention group

	No intervention				Intervention			
	Baseline	15 months	27 months	39 months	Baseline	15 months	27 months	39 months
	*M* (*SD*)	*M* (*SD*)	*M* (*SD*)	*M* (*SD*)	*M* (*SD*)	*M* (*SD*)	*M* (*SD*)	*M* (*SD*)
AOSI baseline raw score	9.08 (5.32) *N* = 26	7.31 (5.83) *N* = 26	–	–	10.04 (4.60) *N* = 28	5.93 (4.05) *N* = 27	–	–
ADOS‐2 total score	–	–	6.32 (6.79) *N* = 25	5.13 (5.03) *N* = 24	–	–	4.13 (3.61) *N* = 23	3.96 (3.68) *N* = 27
MACI infant attentiveness^a^	3.65 (1.29) *N* = 26	4.19 (1.13) *N* = 26	4.21 (1.44) *N* = 24	–	3.39 (1.26) *N* = 28	4.22 (1.05) *N* = 27	4.74 (1.63) *N* = 23	–
MACI caregiver nondirectiveness^a^	3.73 (1.43) *N* = 26	3.92 (1.32) *N* = 26	4.29 (1.73) *N* = 24	–	3.50 (1.48) *N* = 28	4.67 (1.24) *N* = 27	5.00 (1.65) *N* = 23	–
DCMA proportion child initiations	–	–	0.47 (0.20) *N* = 24	0.44 (0.14) *N* = 23	–	–	0.57 (0.23) *N* = 23	0.50 (0.18) *N* = 25
DCMA proportion parent synchrony	–	–	0.46 (0.19) *N* = 24	0.45 (0.10) *N* = 23	–	–	0.48 (0.18) *N* = 23	0.43 (0.14) *N* = 25
MSEL receptive raw score	10.81 (1.86) *N* = 26	15.46 (3.25) *N* = 26	25.48 (6.44) *N* = 25	34.17 (7.53) *N* = 24	10.43 (1.67) *N* = 28	13.81 (1.75) *N* = 27	26.43 (4.41) *N* = 23	34.07 (5.33) *N* = 27
MSEL receptive *T*‐score	50.38 (9.30) *N* = 26	43.04 (12.11) *N* = 26	48.92 (13.68) *N* = 25	50.75 (13.59) *N* = 24	49.89 (9.03) *N* = 28	36.89 (7.13) *N* = 27	50.57 (12.64) *N* = 23	50.30 (12.61) *N* = 27
MSEL expressive raw score	10.73 (1.73) *N* = 26	15.42 (2.93) *N* = 26	24.00 (6.17) *N* = 25	34.54 (7.48) *N* = 24	10.21 (2.17) *N* = 28	14.41 (2.00) *N* = 27	24.87 (4.86) *N* = 23	34.22 (5.58) *N* = 27
MSEL expressive *T*‐score	55.04 (9.99) *N* = 26	49.77 (11.13) *N* = 26	47.36 (13.96) *N* = 25	50.88 (12.35) *N* = 24	53.54 (11.27) *N* = 28	46.52 (8.69) *N* = 27	49.96 (12.23) *N* = 23	50.33 (12.37) *N* = 27
Vineland communication standard score	92.68 (16.18) *N* = 25	98.76 (14.74) *N* = 25	99.76 (16.29) *N* = 25	95.92 (16.47) *N* = 24	91.78 (18.54) *N* = 27	93.42 (11.05) *N* = 26	100.48 (11.97) *N* = 23	98.27 (12.33) *N* = 26
Vineland socialisation standard score	99.92 (10.33) *N* = 25	97.76 (13.11) *N* = 25	98.28 (15.78) *N* = 25	94.54 (13.73) *N* = 24	94.93 (16.26) *N* = 27	99.27 (12.04) *N* = 26	98.04 (10.94) *N* = 23	95.27 (13.80) *N* = 26
Attention disengagement ms	182.79 (81.59) *N* = 25	171.62 (100.39) *N* = 24	163.99 (129.67) *N* = 20	–	209.74 (76.49) *N* = 28	160.84 (81.75) *N* = 26	158.03 (84.90) *N* = 20	–
Diagnostic outcome				*N* (%)				*N* (%)
HR no ASD				16 (62)				16 (59)
HR atypical				8 (31)				7 (26)
HR ASD				2 (8)				4 (15)

a 1 (low) to 7 (high) scoring scale; AOSI, Autism Observational Schedule for Infants; ADOS‐2, Autism Diagnostic Observation Schedule – 2nd Edition; DCMA, Dyadic Communication Measure for Autism; MACI, Manchester Assessment of Caregiver‐Infant Interaction; MSEL, Mullen Scale of Early Learning; Vineland, Vineland Adaptive Behavior Scale standard scores.

### Autism prodromal symptoms

**Figure 2:**
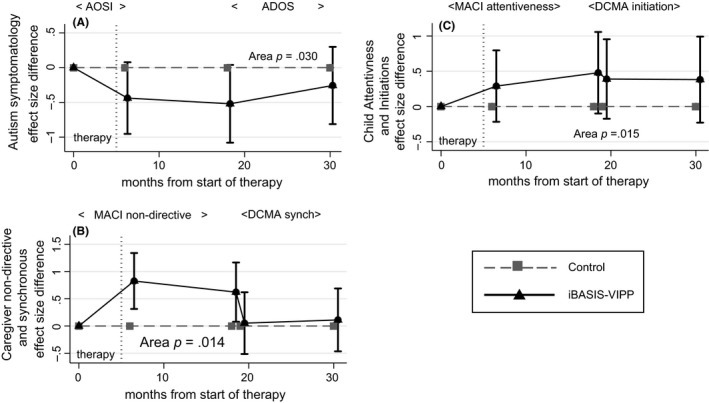
Time profile of treatment effects on autism symptoms and caregiver–child interaction (‘area’ = area between curves estimation; see text). The effect size difference is shown by holding TAU as zero. (A) Primary Outcome, Autism Prodromal Symptoms. (The negative effect size reflects a reduction in symptom severity in iBASIS‐VIPP relative to TAU). (B) Parental Dyadic Social Interaction. (C) Child Dyadic Social Interaction

Figure 2A shows the jointly estimated point treatment effects and CIs at each time point for autism prodromal symptoms. The effects were close to individual significance immediately following intervention (15 months), and showed some evidence of persistence beyond this point and across the change of instrument from AOSI to ADOS‐2. Combining the estimates to form the overall area between curves for the control and iBASIS‐VIPP groups from the start of therapy to 24 months after end of therapy showed a significant ES in favour of intervention of 0.32 (95% CI 0.04, 0.60; *p *=* *.03). Analysis of the effect on autism prodromal symptom scores of dropping case‐wise (Figure S2) suggests that this treatment effect is not driven by outliers or a subset of cases.

### Diagnostic outcome

The intervention group showed 4/27 (15%) children with clinical ASD outcome, 7/27 (26%) with atypicality and 16/27 (59%) typical development. Nonintervention showed 2/26 (8%) ASD, 8/26 (31%) non‐ASD atypical and 16/26 (62%) typical development (Table 1). There was no intervention effect on autism diagnostic outcome (2 by 3 Fisher's exact *p *=* *.846; ordinal logistic OR = 0.83, *p *=* *.726).

### Dyadic interactions

For parental dyadic behaviours (Figure 2B), the strong early effects of intervention on parental nondirectiveness/synchrony begin to reduce by 27 months, but with a marked further reduction on switching from the MACI to the DCMA measure, for which no persisting effect of intervention is evident. Nonetheless, the large early effects are sufficient to yield a significant overall treatment effect on parental interactive behaviour of 0.33 (95% CI 0.04, 0.63; *p *=* *.013). Measures of child dyadic social interaction (Figure 2C) are notably consistent across time and measure, with little evidence of attenuation; the overall ES was significant at 0.36 (95% CI 0.04, 0.68; *p *=* *.015). Once again, there was no suggestion that outliers or a subset of cases drove these effects (Figures S2b and S2).

### Developmental measures

**Figure 3:**
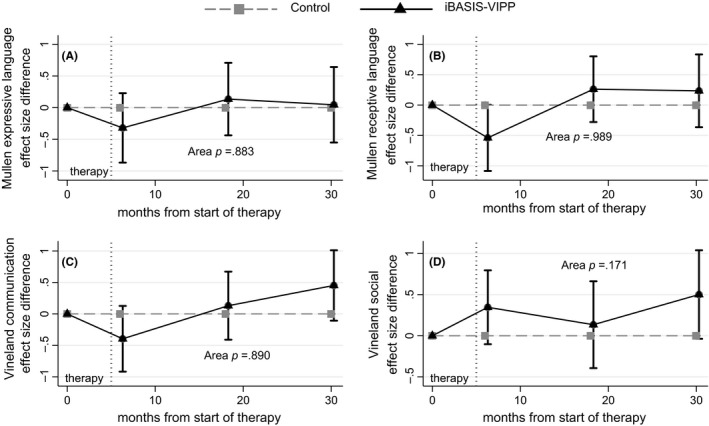
Time profile of effects on Mullen language and Vineland communication and social scores (area = area between curves estimation; see text)

Both Mullen language measures and the Vineland communication measure show a nonsignificant trend towards initial slowing in the intervention group followed by a delayed trend towards benefit (Figure 3). The overall effects over time are all nonsignificant (all *p *>* *.88). For the (nonblinded) parent‐report Vineland social scale, the longitudinal data have not clarified the initial suggestive early nonsignificant gains. While estimates remain positive at both 27 and 39 months, they lack sufficient stability to yield a significant overall effect (area *p *=* *.171).

## Discussion

We report on the follow‐up to age 3 years of the first randomised trial of a pre‐emptive intervention with infants at high familial risk of developing autism. The intervention is of relatively moderate intensity compared to some autism interventions (mean 9.5 sessions/family, range 6–11; Green et al., 2015); meta‐analysis of similar parent–infant psychosocial interventions in nonautism contexts finds generally better results from briefer interventions (Bakermans‐Kranenburg, van IJzendoorn, & Juffer, 2003). The planned follow‐up used a repeated measures design to analyse the developmental trajectories for prespecified outcomes.

For autism prodromal symptom severity, point estimates of treatment effect reach a maximum at 27 months (10 months after treatment end) and were less by 39 months. At each individual time point, there was a lack of precision and 95% CIs include the null. However, the averaged combined estimate over time (using area under curve) shows a significant positive overall treatment effect. We thus demonstrate, to our knowledge for the first time, that a very early intervention for at‐risk infants has produced a sustained alteration of subsequent child developmental trajectory; reducing prodromal autism symptoms into the second and third years of life to a total of 24 months following end of the intervention.

Post hoc investigation of the developmental and clinical significance of this result first suggests a general effect across the whole cohort rather than on a specific subgroup (Figures S2). This is consistent with the intervention targeting early parent–infant developmental interaction processes that are generic across typical and atypical early development: although they show specific perturbation (and thus theoretically a need for enhanced developmental support) in this at‐risk group. Second, while there was no formal typical development comparison group within the trial, post hoc indexing of AOSI and ADOS score severity in relation to ‘low‐risk’ (LR) infants with no familial risk of autism within the longitudinal project from which the sample was drawn (*n* = 27), suggested that baseline symptom scores in the HR trial cohort were raised relative to LR infants, and at follow‐up endpoint, while the nonintervention group remained elevated, the intervention group had reduced ADOS scores into the LR range (for details see Supporting Information).

The study was not powered to test for a treatment effect on categorical autism outcome, and there is no evidence for such an effect in the data. Trials in larger cohorts would be necessary to give the power to test whether improving these prodromal symptoms across the cohort could ever affect the amount of categorical ASD outcome. The measurement of categorical outcome is through a clinical best estimate from triangulating multiple sources of information, whereas that of the prodromal symptoms relates to a continuous measure of severity on one instrument. We do not have the data to inform whether the lack of consistency between measured effect on categorical outcome and symptom severity relates to such measurement issues or sample size.

The parent‐mediated iBASIS‐VIPP intervention worked with parents to increase their awareness and timing of response to infant communications. The success of this strategy in iBASIS is marked by the initial increase in parental ‘nondirectiveness’ within dyadic interaction (Green et al., 2015; Figure 2B). Other early interventions using similar structured video‐aided techniques have shown improvements in the same or related parental interaction behaviours, in both autism (Green et al., 2010; Kasari et al., 2014; Poslawsky et al., 2015; Rahman et al., 2016) and nonautism (Juffer et al., 2008) contexts. This gives persuasive support for the efficacy of these video‐aided methods and may relate to the less predictable effect in parent interventions that do not use this approach. The fall off in parental change during follow‐up may suggest a value for phased ‘booster sessions’ for families in the second or third years. Formal mediation tests in two related studies (Aldred et al., 2012; Pickles et al., 2016) show that such parental synchrony change mediates child symptom changes. A larger sample would have been necessary to test such mediation in iBASIS, but this study does contribute follow‐up data that show, for the first time to our knowledge in an infancy study, a sustained intervention effect from parent‐mediated intervention on child dyadic communication and symptom outcomes, extending over 24 months after treatment end. The intervention targeted precursors of social competency (Tomasello, 2008), shown to be disrupted in the autism prodrome (Wan et al., 2013); the success of this intervention strategy suggests therefore that, in this respect, autism atypicality is responsive to similar processes as in neurotypical development. We reported at endpoint (Green et al., 2015) a possible trend towards negative impact of the intervention on early language development and auditory neural sensitivity. This is not seen in the follow‐up data, where the pattern across both assessed and reported language measures does not suggest any effect (Figure 3A–C).

### Methodological issues

The ‘area‐between‐therapies’ analysis over time used here can be an efficient way to assess the cumulative benefits of intervention in developmental treatments (Pickles et al., 2016). In this paper, we illustrate an extension of this approach to a common challenge in early development, where the assessment instruments on which treatment differences are measured change as children grow older during the trial. Our findings also have implications for the design of future trials from infancy where blind‐assessment often necessitates video‐coding of interaction. With repeated assessments we are able to reduce the variance due to measurement error, increasing reliability and thus statistical power, leading to greater clarity of findings and hopefully replicability.

### Strengths and limitations

The study used a manualised intervention, targeting aspects of early development associated with later autism (dyadic interaction and autism prodromal symptoms) and embedded in a closely studied longitudinal cohort. It achieved a high rate of participant follow‐up and completion of blind‐rated assessments, within a prespecified, hypothesis‐driven analysis that made efficient use of the repeated measurement. A weakness of the study was the relatively modest sample size, which reduced precision of estimates and precluded mechanism analysis. Also, so as to be developmentally appropriate across the age span from 9 months to 3 years, we had to vary measures on key domains between time points, although we mitigated this with the measurement design and statistical approach used. This was a ‘selective’ pre‐emptive trial in an at‐risk group not selected for developmental atypicality; about two thirds of the overall cohort were typically developing at age 3 years with 6/53 (11%) showing categorical ASD. Similar experimental intervention design with ‘indicated’ sampling (i.e. showing actual early atypicality) might give different results.

**Key points**

- There have been no previous trials of pre‐emptive intervention in the infancy prodrome of autism reporting behavioural outcomes; the few studies in the second year have had mixed results.
- This first RCT of a pre‐emptive intervention in the first year sampled infants at familial risk. It undertook repeated measures assessment from the end of treatment (15 months) to 27‐month and then 39‐month follow‐up.
- The 5‐month parent‐mediated video‐aided intervention (9–14 months) reduced the severity of subsequent prodromal autism symptoms over the period to 39‐month follow‐up, as well as producing positive impact on dyadic parent–infant interactions.
- These findings are encouraging for the possibilities of pre‐emptive intervention in autism. Future trials could be powered so as to identify mechanisms of effectiveness and test further questions within autism developmental science.

## Supporting information


**Appendix S1.** Participants.
**Appendix S2.** Allocation and masking.
**Appendix S3.** Further details on intervention.
**Appendix S4.** Further details on measures.
**Appendix S5.** Further details of statistical analysis.
**Appendix S6.** Intervention within the British Autism Study Of Infant Siblings (i‐Basis).
**Appendix S7.** Parent–child interaction measurement – Coding domains and operationalisation on Manchester Assessment of Caregiver–Child Interaction (MACI‐Infant and MACI‐Toddler) and Dyadic Communication Measure for Autism (DCMA).
**Appendix S8.** CONSORT checklist.
**Table S1.** Baseline characteristics of the Intervention and Nonintervention groups.
**Table S2.** Correlations over time and between measures in the selected domains.
**Figure S1.** Effect size estimates for the primary and secondary outcomes at 27 months.
**Figure S2.** Distribution of intervention effect across the cohort.
