# Supplementary material for: Randomised trial of a parent‐mediated intervention for infants at high risk for autism: longitudinal outcomes to age 3 years
Source: J Child Psychol Psychiatry. 2017 Apr 10;58(12):1330–40. doi: 10.1111/jcpp.12728 (PMC5724485; doi:10.1111/jcpp.12728)
Supplement: Supplementary file 1 — Appendix S1. Participants. Appendix S2. Allocation and masking. Appendix S3. Further details on intervention. Appendix S4. Further details on measures. Appendix S5. Further details of statistical analysis. Appendix S6. Intervention within the British Autism Study Of Infant Siblings (i‐Basis). Appendix S7. Parent–child interaction measurement – Coding domains and operationalisation on Manchester Assessment of Caregiver–Child Interaction (MACI‐Infant and MACI‐Toddler) and Dyadic Communication Measure for Autism (DCMA). Appendix S8. CONSORT checklist. Table S1. Baseline characteristics of the Intervention and Nonintervention groups. Table S2. Correlations over time and between measures in the selected domains. Figure S1. Effect size estimates for the primary and secondary outcomes at 27 months. Figure S2. Distribution of intervention effect across the cohort. [file JCPP-58-1330-s001.docx]

**Supporting information for article entitled:**

***Randomised trial of a parent-mediated intervention for infants at high risk for autism: longitudinal outcomes to age three years***

**by Green et al. (2017)**

Green J., Pickles A., Pasco G., Bedford R., Wan MW., Elsabbagh M., Slonims V., Gliga T., Jones EJH., Cheung CHM., Charman T., Johnson MH., and the British Autism Study of Infant Siblings (BASIS) Team.

**Appendix S1.** Participants.

*High risk infants*

Families were recruited via the British Autism Study of Infants Siblings (BASIS) network (<http://www.basisnetwork.org>). All 54 high-risk infants had an older sibling (hereafter, proband; 50 boys, 4 girls) with a community clinical diagnosis of ASD. For 52 of the 54 probands parents had completed the Development and Wellbeing Assessment (DAWBA; Goodman, Ford, Richards, Gatward, & Meltzer, 2000) and/or the Social Communication Questionnaire (SCQ; Rutter, Bailey & Lord, 2003). Thirty-six probands met criteria on both the DAWBA and SCQ. While a small number scored below threshold on the SCQ (n = 4) no exclusions were made, due to meeting threshold on the DAWBA and expert opinion. For 11 probands, confirmation of local clinical diagnosis was only available for the SCQ and in one proband this was only available for the DAWBA. For 2 probands neither measure was available aside from parent-confirmed community clinical ASD diagnosis.

*Low risk comparison*

For comparison we also report AOSI and ADOS scores for a group of low risk controls who were not part of the RCT. Infants in the LR control group were recruited from a volunteer database at the Birkbeck Centre for Brain and Cognitive Development (CBCD). Inclusion criteria included full-term birth, normal birth weight, and lack of any ASD within first-degree family members (as confirmed through parent interview regarding family medical history). All LR participants had at least one older sibling.

**Appendix S2.** Allocation and masking.

Participants were enrolled through trial administrators at Birkbeck College and The University of Manchester. Recruitment took place between April 2011 and December 2012 and follow-up at 27 months during 2012 and 2014 and at 39 months from 2013 to 2015. After consent and baseline assessment, family details were registered at the Manchester trial office and identification number and centre telephoned to an independent statistician at the Christie Clinical Trials Unit in Manchester. Allocation was by individual randomisation 1:1 to intervention or no-intervention stratified by centre (London, Manchester) using a permuted block approach within the two strata with random block sizes of four or six and generated by the Clinical Trials Unit statistician. Assessors and supervising research staff were independent from therapists, were housed in different buildings and were blind to intervention allocation and method of randomisation. Intervention allocation could not be masked from families and therapists. All assessments were administered and coded blind to other information including group allocation, with the exception of parent-rated measures of adaptive behaviour.

**Appendix S3.** Further details on intervention.

The nature of iBASIS intervention was designed with two key considerations. Firstly that it had to be suitable for infants and dyads who would not go on to develop autism (since this was an at risk group within a selective prevention trial and at least half of whom were expected to proceed to typical outcomes). It would need therefore to be an intervention that had been implemented within typically developing children without adverse effect. Secondly, since the core defining impairment in autism is one of social understanding and reciprocity, the target should be the foundational social processes underlying these skills. While there are enduring debates about the nature of these skills, in this intervention we targeted a range of different processes, e.g. parent contingent responding, social reciprocity, intersubjectivity and shared affect (Tomasello, 2008). Furthermore the interactive specialisation model of functional brain development (Johnson, 2011) would suggest that those socially expectant aspects of the neural system underlying social development might be critically sensitive to social enrichment of this kind at this time.

In home based sessions, the therapist makes a video-tape of interactions between parent and child and uses video excerpts in a series of developmentally sequenced sessions to improve the quality of parent understanding of infant’s communication. The focus sequence comprises: interpreting the infant’s behaviour and recognising their intentions, enhancing sensitive responding, emotional attunement and patterns of verbal and non-verbal interaction. Because of the developmental complexity of prodromal ASD we extended the original VIPP program from six sessions by adding up to six planned booster sessions according to need and in discussion with family, and added therapeutic procedures to address any emerging developmental atypicality (intervention protocol available from the authors and online). Therapists were a graduate speech and language therapist and a doctoral level psychologist, trained and supervised at two centres (one at each site in Evelina Children’s Hospital, London and The University of Manchester). Therapist fidelity to the manual was assessed on twenty-three sessions from 15 participants, randomly selected to balance time-point and therapist, and double-coded using a 21 pass/fail item measure of specific iBASIS-VIPP strategies and therapeutic skills. Mean fidelity score was 19.4/21 (93%; range 15-21), with only 1/23 sessions below the pre-specified 80% fidelity threshold.

All 27 families who began treatment (12 in Manchester, 15 in London) completed the six core sessions over 5 months, with a mean of 9.5 of 12 possible sessions attended per family (SD 1.6, range 6–11). No adverse effects from intervention were reported.

The comparator group had no planned intervention. Within the UK, context specific interventions for familial at-risk siblings in the second year of life would be sparse or non-existent. This needs to be understood in the context that the majority of the at-risk siblings were typically developing and few had a community clinical diagnosis of a developmental disorder. Only one parent (in the non-intervention group) reported that their child was in receipt of a specific intervention (1 hour per week of speech and language therapy) between the 15 month and 27 month assessment. By 39 month follow up, eight children from the treatment group and thirteen from the no treatment group were reported to have accessed services by the 39 month visit. Of these, four treatment group and eight no treatment group children attended community-based activities (such as open drop-in sessions, ‘music-and-interaction’ and other non-specialist groups) only. Regarding services specifically for children with additional needs (including early intervention, speech and language therapy, occupational therapy, physical therapy and services for children with developmental delay); three children from the treatment group and one from the no treatment group accessed one or two of these services, and three children from each group accessed three or more. In most cases the intensity was low (most commonly one hour per week) with the exception of one child from the intervention group who received 12 hours per week of one-to-one special education support in nursery; two children from the no intervention group who received 12 and 15 hour per week, respectively, of special education support and one of these children in addition was receiving 12 hours per week of behavioural intervention.

**Appendix S4.** Further details on measures.

Manchester Assessment of Caregiver-Infant Child interaction (MACI) was designed to investigate interactional antecedents of social competency and early parent-infant interaction in the autism prodrome. An infancy version was used at the 9 and 15 month assessments, and toddler version at 27 months.

*Clinical ASD outcome*

From 54 initially randomised participants: one could not be classified due to lack of both 27 and 39 month follow-up data; 6 (11%; all boys) met criteria for ASD (American Psychiatric Association, 2013); 15 (28%; 10 boys, 5 girls) were non-ASD but categorised as ‘Atypical’ due either to: a) meeting ADOS criteria, with or without ADI-R (Risi et al., 2006) (*n* = 6); b) scoring greater than 1.5 SD below the population mean on the Mullen ELC (< 77.5) or on the Mullen EL or RL subscales (< 35) (*n* = 5); or c) both criteria a) and b) (*n* = 4). The remaining 32 participants (59%; 12 boys, 20 girls) were considered typically-developing. We note that whilst assignment to an ASD diagnosis is a clinical judgement based on all available evidence against DSM-5 criteria, the ‘atypical’ category is instrumentally defined to separate these children from high-risk children who are incontrovertibly typically developing. This categorization has been widely used in reporting outcomes at age 3 years both by our group and by many others who study high-risk siblings (Cheung et al., in press; Hudry et al., 2014; Ozonoff et al., 2014), including in consortium publications by the BSRC (Chawarska et al., 2014).

**Appendix S5.** Further details of statistical analysis.

As described in the main paper, combined analysis of the treatment effect estimates available at trial 15, 27 and 39 months used Seemingly Unrelated Regressions (Zellner, 1962) estimated by maximum likelihood using the *sem* procedure using the mlmv option. The mlmv method option uses a full-information maximum likelihood estimator in which records with missing outcomes are included in the analysis and treated under an assumption of missing at random. All scores were untransformed except measures of autism symptomatology where a skew reducing transformation of log(k+score) was applied, where k=1 for all post-baseline measures. The pairwise correlation among adjacent transformed AOSI/ADOS measures were modest, the largest being 0.37, and declined with increased separation to 0.14. Measures of child dyadic social interaction (inevitably obtained from brief episodes of infant-caregiver interaction) also modest (Supplementary Table 2), the highest correlations being 0.25 and 0.27, achieved between the baseline MACI and 27 month DCMA and between the two DCMA measures respectively. For parental dyadic behaviours Supplementary Table 2 shows the cross-measure correlations as high as those within-measure, though again overall the correlations are of modest size. Supplementary Table 2 shows moderate correlations across time in the Mullen expressive and receptive T-scores, and the Vineland communication and social standard scores. An exception is the weak correlation of the baseline assessment of receptive language with the later measurements. The joint analysis of measures at 39 months with those of 15 and 27 months took account of these correlations to provide effect estimates for each post-baseline measurement.

For the transformed autism symptom measures we estimated three correlated regressions for AOSI total at 15 months, and ADOS-2 total at 27 and 39 months, covarying each for baseline AOSI, centre, age-at-assessment, mother’s ethnicity and educational qualifications (imbalanced at follow-up), treatment group assignment, and at 39 months a dummy variable for the ADOS module used; all Module 1 at 27 months, and 10 Module 1 and 41 Module 2 at 39 months (no intervention: 4 Module 1, 20 Module 2; intervention: 6 Module 1, 21 Module 2).

For *child interaction* we used MACI infant attentiveness at 15 and 27 months, and DCMA proportion child initiations at 27 and 39 months, covarying each for baseline MACI infant attentiveness, centre, age-at-assessment, mother’s ethnicity and educational qualifications and treatment group assignment.

For parent interaction we used MACI caregiver non-directiveness at 15 and 27 months, and DCMA proportion caregiver synchronous behaviours at 27 and 39 months, covarying each for baseline MACI caregiver non-directiveness, centre, age-at-assessment, mother’s ethnicity and educational qualifications and treatment group assignment.

For Mullen expressive and receptive T-scores at 15, 27 and 39 months we covaried for baseline Mullen score, centre, age-at-assessment, mother’s ethnicity and educational qualifications and treatment group assignment. For Vineland social and communication standard scores at 15, 27 and 39 months we covaried for baseline Vineland score, centre, mother’s ethnicity and educational qualifications and treatment group assignment.

*Unplanned tests*

1) An additional unplanned statistical diagnostic test to examine the degree to which the principal finding of an overall treatment effect as measured by the AOSI and ADOS was undertaken by calculating the percentage change in the effect size estimate on leaving out each of the 54 participants.

2) Post-hoc indexing of AOSI and ADOS score severity in relation to infants within the longitudinal project from which the sample was drawn who had no familial risk of autism. AOSI scores in this low risk (LR) group (n=27) at 9 month baseline showed a mean score of 5.26 (SD 3.1) and at 15 months mean 4.07 (SD 3.5). This compares to the equivalent means in our cohort of 9.08 (non-intervention) and 10.04 (intervention) at baseline and 7.31 and 5.93 respectively at 15 months (Table 1); suggesting that the HR group in this trial had raised baseline symptom scores compared to LR controls. ADOS scores in the LR group at 39 month endpoint, showed mean 3.68 (SD 3.1), comparable to the iBASIS intervention group (mean 3.96), and in contrast to the non-intervention which remained elevated (mean 5.13). The inference is that the intervention has acted towards reducing ADOS scores into the LR range by end of follow-up.

**References**

American Psychiatric Association. (2013). *Diagnostic and Statistical Manual of Mental Disorders*, 5th Edition: DSM-5.

Chawarska, K., Shic, F., Macari, S., Campbell, D.J., Brian, J., Landa, R., Hutman, T., Nelson C, A., Ozonoff, S., Tager-Flusberg, H., Young, G.S., Zwaigenbaum, L., Cohen, I. L., Charman, T., Messinger, D.S., Klin, A., Johnson, S., & Bryson, S. (2014). [18-month predictors of later outcomes in younger siblings of children with autism spectrum disorder: a baby siblings research consortium study.](https://www.ncbi.nlm.nih.gov/pubmed/25457930) *Journal of the American Academy of Child & Adolescent Psychiatry*, *53*(12), 1317-1327.

Cheung, C. H. M., Bedford, R., Johnson, M. H., Charman, T., & Gliga, T. (2016). Visual search performance in infants associates with later ASD diagnosis. *Developmental Cognitive Neuroscience*. doi: 1.1016/j.dcn.2016.09.003.

Goodman, R., Ford, T., Richards, H., Gatward, R., & Meltzer, H. (2000). The Development and Well-Being Assessment: description and initial validation of an integrated assessment of child and adolescent psychopathology. *Journal of Child Psychology and Psychiatry*, *41*(05), 645-655.

Hudry, K., Chandler, S., Bedford, R., Pasco, G., Gliga, T., Elsabbagh, M., Johnson, M.H., & Charman, T. (2014). [Early language profiles in infants at high-risk for autism spectrum disorders.](https://www.ncbi.nlm.nih.gov/pubmed/23748385) *Journal of Autism and Developmental Disorders*, *44*(1), 154-167.

Johnson, M. H. (2011). Interactive specialization: a domain-general framework for human functional brain development? *Developmental Cognitive Neuroscience*, *1*(1), 7-21.

Ozonoff, S., Young, G.S., Belding, A., Hill, M., Hill, A., Hutman, T., Johnson, S., Miller, M., Rogers, S.J., Schwichtenberg, A.J., Steinfeld, M., & Iosif, A.M. (2014). The broader autism phenotype in infancy: when does it emerge? *Journal of the American Academy of Child & Adolescent Psychiatry*, *53*(4), 398-407.Rutter, M., Bailey, A., & Lord, C. (2003). *The social communication questionnaire: Manual*. Western Psychological Services.

Tomasello, M. (2008). *Origins of human communication*. Cambridge, Massachusetts: The MIT Press.

Zellner, A. (1962). An efficient method of estimating seemingly unrelated regressions and tests for aggregation bias. *Journal of the American statistical Association*, *57*(298), 348-368.

|  | **No Intervention**  **N = 26** | **Intervention**  **N = 28*** |
| --- | --- | --- |
| **Maternal medical history** No disorder  Mental/physical disorder**^a^** | 15 (57.7%)  11 (42.3%) | 17 (60.7%)  11 (39.3%) |
| **Maternal ethnicity**  Caucasian  Other | 22 (84.6%)  4 (15.4%) | 18 (64.3%)  10 (35.7%) |
| **Maternal qualifications^*^**  > Degree  < Degree | 15 (57.7%)  11 (42.3%) | 10 (37.0%)  17 (63.0%) |
| **Annual household^*^ income**  < £40,000  > £40,000 | 15 (57.7%)  11 (42.3%) | 16 (59.3%)  11 (40.7%) |
| **Sex**  Male  Female | 12 (46.2%)  14 (53.8%) | 17 (60.7%)  11 (39.3%) |
| **Typical older sibling(s)**  TD sib(s)  No TD sib(s) | 15 (57.7%)  11 (42.3%) | 14 (50.0%)  14 (50.0%) |
| **Age/days**  Mean (SD)  **MSEL Nonverbal T-score** Mean (SD) | 276.58 (24.25)  57.29 (10.69) | 267.14 (20.93)   - 1. (12.73) |

**Table S1**. Baseline characteristics of the Intervention and Non-Intervention groups.

Data are n (unimputed sample %) or mean (SD) for available cases.

TD = typically developing; MSEL = Mullen Scales of Early Learning.

* N = 27 for Maternal qualifications and annual household income.

a The most commonly reported conditions were depression (*n* = 15), anxiety disorder (*n* = 4), medical conditions (e.g. seizures, sickle cell) (*n* = 3).

**Table S2.** Correlations over time and between measures in the selected domains.

|  | **Autism symptoms** | | |  |
| --- | --- | --- | --- | --- |
|  | AOSI  **15m** | ADOS2  **27m** | ADOS2  **39m** |  |
| AOSI **9m** | .32 | .21 | .16 |  |
| AOSI **15m** |  | .29 | .14 |  |
| ADOS **27m** |  |  | .37 |  |
|  | **Child attentiveness/initiations** | | | |
|  | MACI Att  **15m** | MACI Att  **27m** | DCMA Init  **27m** | DCMA Init **39m** |
| MACI Att **9m** | .06 | .12 | .25 | -.14 |
| MACI Att **15m** |  | .12 | -.04 | -.08 |
| MACI Att **27m** |  |  | .16 | -.08 |
| DCMA Ini **27m** |  |  |  | .27 |
|  | **Parent non-directiveness/synchrony** | | | |
|  | MACI N-D **15m** | MACI N-D  **27m** | DCMA Sync  **27m** | DCMA Sync  **39m** |
| MACI N-D **9m** | .08 | .19 | .18 | .18 |
| MACI N-D **15m** |  | .34 | .09 | .20 |
| MACI N-D **27m** |  |  | .32 | .18 |
| DCMA Sync **27m** |  |  |  | .22 |
|  | **MSEL Expressive language T score** | | | |
|  | **15m** | **27m** | **39m** |  |
| Exp-T **9m** | .25 | .15 | .09 |  |
| Exp-T **15m** |  | .56 | .46 |  |
| Exp-T **27m** |  |  | .72 |  |
|  | **MSEL Receptive language T score** | | |  |
|  | **15m** | **27m** | **39m** |  |
| Rec-T **9m** | .00 | .25 | .17 |  |
| Rec-T **15m** |  | .48 | .39 |  |
| Rec-T **27m** |  |  | .74 |  |
|  | **Vineland Communication** | | |  |
|  | **15m** | **27m** | **39m** |  |
| Com **9m** | .39 | .21 | .29 |  |
| Com **15m** |  | 0.57 | .54 |  |
| Com **27m** |  |  | .86 |  |
|  | **Vineland Socialisation** | | |  |
|  | **15m** | **27m** | **39m** |  |
| Soc **9m** | .58 | .46 | .34 |  |
| Soc **15m** |  | .45 | .48 |  |
| Soc **27m** |  |  | .72 |  |

**Figure S1.** Effect size estimates for the primary and secondary outcomes at 27 month.

ASD symptoms = ADOS-2 Total Score

Child initiation=DCMA proportion of interactions that were child initiations

Child Attention=MACI child attention to caregiver

Parent non-directiveness=MACI parental non-directiveness

Receptive language=Mullen receptive language raw-score

Expressive language=Mullen expressive language raw score

Communication=Vineland communication standard score

Socialization=Vineland socialization standard score

Disengagement=Speed of disengagement of visual attention in the Attention Disengagement task

**Figure S2.** Distribution of intervention effect across the cohort

To examine how dependent the estimated treatment effects were to a small number of cases, we re-estimated the models for each of the primary outcomes of interest, dropping each case in turn. We have displayed these results in two ways. The first shows the percentage change (baseline to endpoint) by case-number while the second shows their distribution. The null expectation where the true effect is common to all cases, is that the distribution of these changes in estimates should be approximately normal. The power to discriminate a small sub-group effect is low but the distributions of influence are largely as expected for a common effect.

**Appendix S6.** Intervention protocol (iBASIS-VIPP)

**AUTHORS**

**Samina Holsgrove^1^, Ming Wai Wan^1^, Janet McNally^1^, Rhonda Booth^2^, Carol Taylor^1^,Vicky Slonims^2^, Jonathan Green^1^**

1. **University of Manchester**
2. **Guys and St Thomas’s NHS Foundation Trust/Kings College London**

**ACKNOWLEDGEMENT**

**Much of the procedural manual contained here is based on or adapted from the original work and manual of the Video Interaction to Promote Positive Parenting (VIPP)**

Juffer, F., M.J. Bakerman-Kranenburg, and M.J. Van Ijzendoorm, *Promoting Positive Parenting: An Attachment-Based Intervention*. 2008, New York: Taylor Francis

[**www.leidenattachmentresearchprogram.eu/vipp/welcome/en/**](http://www.leidenattachmentresearchprogram.eu/vipp/welcome/en/)

**AIM**

Enrichment of the social interactive environment of the developing infant using a parent-mediated programme to enhance early social engagement and reciprocity.

**The intervention strategy**

The i-BASIS intervention strategy is a parent-mediated approach to achieve two goals;

1. general enrichment of the core interactive social experience for infants 9-14 months inclusive
2. specific attention within this to addressing any emerging atypicalities that might be expected in prodromal autism at this age and their interactional consequences

The *i*-BASIS programme comprises twelve home based two hourly sessions over a period of 5 months. The programme is individualised to the needs of each dyad but core procedures are taken from the Video feedback Intervention to promote Positive Parenting (VIPP) [1], [www.leidenattachmentresearchprogram.eu/vipp/welcome/en/](http://www.leidenattachmentresearchprogram.eu/vipp/welcome/en/)). We chose this as the basis because its method (video-aided and parent-mediated using a direct work with parent and infant) is similar to that which we have used intensively with preschool children with diagnosed autism (2, [www.manchester.ac.uk/medicine/pact](http://www.manchester.ac.uk/medicine/pact)) and because of its good evidence base across disorders and in neurotypical groups.

There is a preliminary session (baseline/relationship building with parent); followed by six intervention sessions (delivered weekly to fortnightly); each with a theme building on techniques and learning from the previous session. In the six intervention sessions, the first two focus on infant behaviour (with maternal behaviour alluded to indirectly), the second two sessions address maternal behaviour, and the final two sessions examine more complex chains of interaction. The set up of each session is designed to facilitate exploration of specific targeted themes. There are five booster sessions to consolidate learning.

1.Juffer, F., M.J. Bakerman-Kranenburg, and M.J. Van Ijzendoorm, *Promoting Positive Parenting: An Attachment-Based Intervention*. 2008, New York: Taylor Francis Group.

2. Green J. Charman, T,. McConachie, H., Aldred, C., Slomins, V., Howlin, P., Le Couteur, A., Leadbitter, K., Hudrey, K., Byford, S., Barrett, B., Temple, K., MacDonald, W., Pickles, A., and the PACT consortium. (2010). Parent-Mediated Communication-Focused Treatment for preschool children with Autism (PACT); a randomised controlled trial. *The Lancet*, 375(9732), 2152-2160.

**SESSION PLANS**

INTRODUCTORY SESSION

| **Aim:**  Introduction, rapport building, goal-setting, and baseline measurement. | **Video recording**  Six minute parent-infant interaction: free play with toys |
| --- | --- |

SESSION 1 – “INFANT WATCHING”

Theoretical Focus: Sensitive responding

The parent has an opportunity to observe the focus and choice of activity of their infant without interruption which encourages them to recognize the pace of the infant’s exploratory behaviours and to match her own responses accordingly. The parent’s experience of watching her infant may also encourage her to think of him or her as a “thinking” being and help her appreciate the potential positive impact of a timely and sensitive response to her child’s behaviours.

| **Aim:**  Observing and naming infant social interactive behaviour | **Video recording**  Free play interaction (6 mins)  Non-interactive play (2 mins) |
| --- | --- |

SESSION 2: ‘ SPEAKING FOR THE BABY’

Theoretical Focus: Inference of intentionality

The observations made of the first session are discussed in depth with a focus on the endowment of intentionality to the infant. The purpose is to reinforce parental empathy with the infant’s affect state as this forms the basis of a sensitive contingent response. The parent is encouraged to display this understanding back to the infant, “feeling for them”, so that the infant feels understood.

| **Aim:**  Observing infant interactive behaviour in conjunction with exploratory behaviour | **Video recording**  Free play interaction (6 mins) |
| --- | --- |

SESSION 3: ‘SENSITIVITY CHAINS’

Theoretical Focus: Synchrony and contingent responsiveness

Building on the concepts introduced in session 2, the parent is encouraged to respond to a range of infant behaviours and match her responses to that of the infant, thereby increasing synchrony. The identification of sensitivity chains reinforces the parent’s awareness of contingent responsiveness as she demonstrates attunement to her infant’s needs.

| **Aim:**  Encourage parental contingent responsiveness  Particular reinforcement of inter-personal face to face type interactions | **Video recording**  Naturalistic setting of a meal time or snack time (20-30 mins) |
| --- | --- |

SESSION 4: SENSITIVITY CHAINS AT MEALTIMES

Theoretical focus: Contingent responsiveness in everyday situations

This session focuses on generalizing the skills addressed in session 3 to an everyday context in a naturalistic setting to show the parent that skills such as attunement and synchrony with her infant are applicable to every interaction between them.

| **Aim:**  Generalising contingent responsiveness to a naturalistic setting | **Video recording**  Face-to-face ‘songs and rhymes’ interaction (6 mins) |
| --- | --- |

SESSION 5: ‘SHARING FEELINGS’

Theoretical Basis: Affect matching

Session 5 introduces a technique to enhance maternal empathy: inviting the parent to speak as if she herself were the infant. This is carried out using a video clip of face-to-face interaction to encourage affect matching.

| **Aim:**  Encourage affect matching and empathy  Reinforcement of inter-personal interactions, including eye contact | **Video recording**  Free play with toys, to include reading a book together if possible (4 mins)  “Funny Sound Game” (2 mins) |
| --- | --- |

SESSION 6: ”SHARING TALK”

Theoretical Focus: Communication

In this session the mother is assisted to reflect on more subtle aspects of vocal and non-vocal communication in the context of a structured interaction involving book reading. The aim is to support reciprocal vocalisations in a social context with contingent, attuned responses from the parent.

| **Aim:**  Encourage vocal communication and social babble  Reinforcement of interpersonal interactions, including eye contact | **Video recording**  Free play with toys (6 mins) |
| --- | --- |

SESSIONS 7 – 12: REINFORCEMENT AND BOOSTER SESSIONS; FURTHER MANAGEMENT OF ATYPICALITY

***Reinforcement and booster sessions***

The aim of these sessions is to reinforce the parent’s learning and ensure progress in parent-infant synchrony, attunement and communication as the infant rapidly learns new skills. This will sometimes involve a return to earlier themes e.g. ‘infant watching’, observation and sensitivity to the infant’s particular traits and reinforcing synchronous responses.

***Identification of atypicality***

The therapist in the *i*-BASIS study will not have been involved in the baseline assessment. However, during the intervention sessions there will have been adequate time for the therapist to identify any evidence of atypicality in the infant within the therapy context. Appendix 2 shows a checklist of potential atypicalities. It will be used as a aide memoire by the therapist at the end of sessions and rated on a 0-2 rating scale after the introductory session and then the 3^rd^, 6^th^, 9^th^ and final sessions. Identified atypicalities will be discussed with the parent in terms of the infant’s behavioural repertoire *without labelling them as prodromal signs*. They will be identified as potential barriers to the processes of reciprocity and shared communication and appropriate advice will be given to facilitate interaction. The degree of interactional perturbation is likely to vary considerably with each infant and parent. Thus the selection of intervention approaches will be tailored to the individual dyad. The therapist will adopt a collaborative and exploratory approach with the parent to reduce the impact of these potentially atypical behaviours.

We have considered it important to have an intervention that does not assume atypicality in a group of infant siblings of children with autism spectrum disorder. In cases where a parent and infant have successfully established reciprocal and mutually satisfactory interaction within the 6 intervention sessions or before the end of the booster sessions the final visits can be spaced more or the total number limited by mutual agreement with parent. In this way *i*-BASIS has built-in flexibility to the heterogeneity of development in the intervention group. The generic parental enhancement techniques in VIPP have demonstrated applicability across a range of normative parenting styles; the additional components more specific to prodromal autism can adapt the intervention where children are presenting with differences in development.

**Potential Areas of Atypicality and Remediation Strategies**

1. See section 2 of the introduction for discussion of atypicalities with reference to literature – this section includes discussion of possible underlying mechanisms for the atypicalities e.g visual preference and ERP response studies.
2. Remediation strategies are designed to either (a) focus on the ‘interactional perturbations’/ dyadic consequences of the atypicality on the parent-infant interaction and encourage more typical interactions where possible or (b) provide the infant with the optimal parent child interaction to improve the atypicalities in the child. In addition remediation strategies attempt to take into account what is known about possible underlying difficulties i.e. visual preferences, sticky attention etc

| OBSERVED ATYPICALITIES IN INFANT | POTENTIAL DYADIC CONSEQUENCES | REMEDIATION | |
| --- | --- | --- | --- |
|  |  | Core I-BASIS Strategies to Emphasise | Additional strategies |
| GAZE BEHAVIOURS   - Reduced/unusual use of eye contact in face-face interactions - Lack of short gaze e.g. to share reactions and check involvement of parent during toy play - Reduced ability to follow parent gaze - Reduced joint attention behaviours | Parental disengagement  Reduction in gaze initiations/length by parent |  | - Enhance parental observation and monitoring of their infant’s use of eye gaze. - Assist parent to recognise episodes in which eye contact may occur and to respond immediately and contingently with a response which is appropriate to their infant’s tolerance i.e. animated and interesting responses which are not overwhelming |
| ATTENTION BEHAVIOURS   - Difficulties in disengagement and smooth pursuit of attention - Overlong staring at toy - Reduced level of gaze switching during play | - Mistiming of parent responses through adults getting ahead of the child - Parent may become intrusive and attempt to impose an attentional shift on the infant which is likely to be counter-productive | - Enhance further the parent’s skill in observing and matching the infant’s focus and the pace of the interaction | - In later stages of the therapy parents can be taught to assist the infant in shifting attention to a range of toys/situations or transitions in routine care. - Encourage parents to sensitively experiment with strategies for shifting attention e.g. touch, sound, using the baby’s name, moving objects to face level? - These strategies should be introduced in terms of “Lets see if we can get him interested in a few more things”. Therapist needs to be careful to explain the balance between following the child’s focus and encouraging attention shifts |
| ATYPICAL PLAY BEHAVIOURS   - Low interest in sharing toys - Tendency to play alone, removed from parent | - Parent may become intrusive and direct/take control of play in an attempt to interact with the infant; this is likely to be counterproductive | - Encourage parent to allow infant to explore the environment and toys - Encourage parent to attend to infant’s focus of interest without interfering | - Encourage parent to observe what types of play their child enjoys most and what opens up opportunities for interaction – e.g. rough and tumble/physical games. - Encourage parent to learn through observing their child’s individual signals which indicate they are ready to join in (help parents understand parents that these signals may be very subtle or weak) - Encourage parents to observe actively, showing interest in what the child is doing, but waiting and watching for signals that indicate the infant’s readiness for them to sensitively join in. |
| ATYPICAL RECIPROCITY   - Reduced reciprocal social smile - Reduced response to name - Reduced response to social talk from parent - Reduced affect matching - Reduced response to parent attempts to engage infant in play - Reduced affective response to social touch | - Disruption of finely tuned reciprocity that typically develops between parent and infant | - Emphasise core I-BASIS procedures that promote reciprocity e.g. affect matching, imitation? - Encourage parents to imitate their infant’s vocalisations e.g. infant vocalises with ‘a’; parent responds ‘aa’; infant vocalises ‘m’; adult responds ‘mm’. | - Encourage a balance of response and the introduction of novel information that is developmentally appropriate. - Build in non-verbal social anticipation games e.g. peek-a-boo into the more structured part of the treatment, particularly in later stages. |
| AFFECT   - Reduced expression of affect - Reduced affect matching | - Parent may miss subtle expressions of affect and not respond appropriately - Lack of response to affect from infant may have led to parent reducing the sharing of their own affect. | - Emphasise the ‘talking for the child’ technique to enhance the parent’s skills in inferring intentionality and affect in the infant. - Assist the parent to recognise infant’s range and unique expression of affect in free play and structured settings. - Encourage the parent to respond to infant’s affect as communicative, interpreting meaning based on contextual cues. - Encourage the parent to mirror affect sensitively whilst monitoring the infant’s response. Establish ways for the parent to reflect back to the infant their understanding; ‘feeling for them’ | - Encourage parents to share their affect responses with the infant. However avoid over exaggeration – work with parent’s natural style - Encourage parents to ensure that the baby has a good view of their face. - Encourage parents to use facial expression when interacting with their baby. - Encourage parents to use expression in their voice when speaking to the baby. |
| EMERGING ATYPICAL COMMUNICATION   - Reduced response to communicative gesture - Reduced use of communicative gestures - Reduced use of protodeclarative pointing - Delays in sound production - Reduced simple and complex babbling - Delays in early word production | - Parent’s may miss some of the infant’s weaker communicative signals and not respond reciprocally; this results in reduced experience of reciprocity for the infant and reduces their language learning opportunities. - Parent’s may become more didactic in their approach to communication, may resort to attempts to ‘teach’ words | - Emphasise I-BASIS procedures for close observation of the focus and intent of the infant - Help parent respond to any vocalisations in a social context that recognises the infant’s intent and underlying affect e.g. infant squeals with joy vocalising ‘a’; adult recognises direction of infant gaze and notices the bin lorry; parent responds with matched vocal pitch ‘lorry’, signalling shared excitement. - Encourage parent to make comments that relate directly to infant’s focus of interest; for instance if the infant throws a toy share and comment on that activity rather than attempting to direct the infant to ‘use the toy properly’. | - Encourage parent to use simple natural gestures and pointing - Encourage the parent to introduce sound games. - Encourage parents to use symbolic sounds. - Assist the parent to provide the infant with developmentally appropriate language models i.e. relating to present context and about items that are visible. - Help parents to recognise their child’s attempts at early word approximations and provide the relevant word – e.g. ‘o’ – ‘dog’. |
| ATYPICAL REACTIVITY AND SENSORY BEHAVIOURS   - Sensitivity to e.g. foods textures, sounds etc - Extremes of temperament – overly reactive or overly passive. | - Extreme and unexpected reactions may adversely affect interaction if parents fail to recognise the reasons for the reaction e.g. may blame themselves for the breakdown in the interaction. - Extreme passivity may result in parents becoming less motivated to interact with their child |  | - Help parent to recognise when atypical reactions are affecting the interaction and to identify possible causes of these.      - Parents will be assisted to experiment with ways to respond to atypical reactions. This may involve assisting the parent to ‘contain’ extreme emotional reactions by the infant with sensitive and well matched affect. - Assist parent to provide support and containment of the infant’s affect state e.g. waiting and remaining engaged while the infant recovers from a particular emotional state rather than attempting to ‘distract’ or move the infant on before they are ready. |
| REPETITIVE BEHAVIOURS   - Atypical motor mannerisms e.g. arm waving | - Parents may monitor for presence of mannerisms and if signs emerge this may lead to parental anxiety which impacts on interaction with child. - Parent may copy mannerisms in a non-social way and/or may use them to initiate interaction. |  | - Encourage parents to consider what the mannerism indicates – e.g. over or under stimulation and respond accordingly - Where possible encourage parent to comment on child’s perceived emotional state e.g. hand flapping through excitement – ‘Oh it’s exciting!’ - Encourage parent to experiment with using imitation as a means of engagement. - Encourage parent to adapt mannerisms into meaningful actions e.g. shaking a shaker, tapping a drum. |

**Appendix S7.** Parent-Child Interaction measurement – Coding domains and operationalization on Manchester Assessment of Caregiver-Child Interaction (MACI-Infant and MACI-Toddler) and Dyadic Communication Measure for Autism (DCMA).

| Domain | Brief description |
| --- | --- |
| **MACI** |  |
| Caregiver | |
| Nondirectiveness *(highly directive to highly nondirective 1-7 scale)* | A behavioural and mental focus on or ‘acceptance’ of the child’s experience, as demonstrated by a lack of demanding, intrusive, and negative behaviours and comments that explicitly or implicitly function to elicit a particular outcome in the child’s behaviour, thus restricting the child’s autonomy. Interactions tend to become more structured as the child develops, and only behaviours that limit or deny the child’s experience are considered in this rating.  Example ratings:  2. Substantial directiveness restricts child contribution to interaction; typically, many (even if moderate) directive behaviours throughout that tend to involve multiple strategies.  3. Directive behaviours result in questionable autonomy; typically, quite frequent examples, resulting in restrictive play.  4. Occasional impact on child autonomy; generally nondirective and accepting of the infant’s experience, with typically several moderate directive behaviours. |
| Child | |
| Attentiveness to caregiver *(inattentive to very highly attentive 1-7 scale)* | Interest in the caregiver (as opposed to focus on other stimuli or self-absorption) as evidenced by (voluntary) positioning, behaviour and communication, including eye contact, speech and vocalisations, acceptance of and interest in caregiver, joint activity, joint attention, face/body orientation, and imitation. As the child develops, interactions become more socially sophisticated, and more consideration is given to the quality of the attentiveness; e.g. affective displays to caregiver during joint activity.  Example ratings:  2. A very slight interest; typically, 2-3 momentary examples, all lacking intensity, such as brief glances or accepting toys which they were earlier disinterested in.  3. Some interest in the caregiver but interest elsewhere is much more apparent; typically, a few moderate examples (or several in toddlers) which are not sustained or may be low-key  4. Clear interest in involving the caregiver but which may be low-level for substantial parts; typically, several examples or up to 3 longer episodes |
| **DCMA** |  |
| **Caregiver** |  |
| Parent synchrony | Parent synchronous acts are utterances that acknowledge, confirm or reinforce the child’s focus, play, actions, thoughts or intentions. To be synchronous the parent’s utterance must be in tune with what the child is thinking, saying or doing and must not attempt to redirect the child away from his play, thoughts or communication or make demands on the child. |
| Child |  |
| Child Initiations | Child non-verbal or verbal communication acts, which start an interaction, are coded as Initiations. A communicative initiation might be signalled by non-verbal behaviours, e.g. showing a toy to the parent, or pulling the parent's hand, or by verbal behaviours e.g. saying “ball” or “more”. |
|  | *Note – DCMA codings are proportion of parent communications that are synchronous and child communications that are initiations (see text)* |

**Appendix S8.** CONSORT checklist.


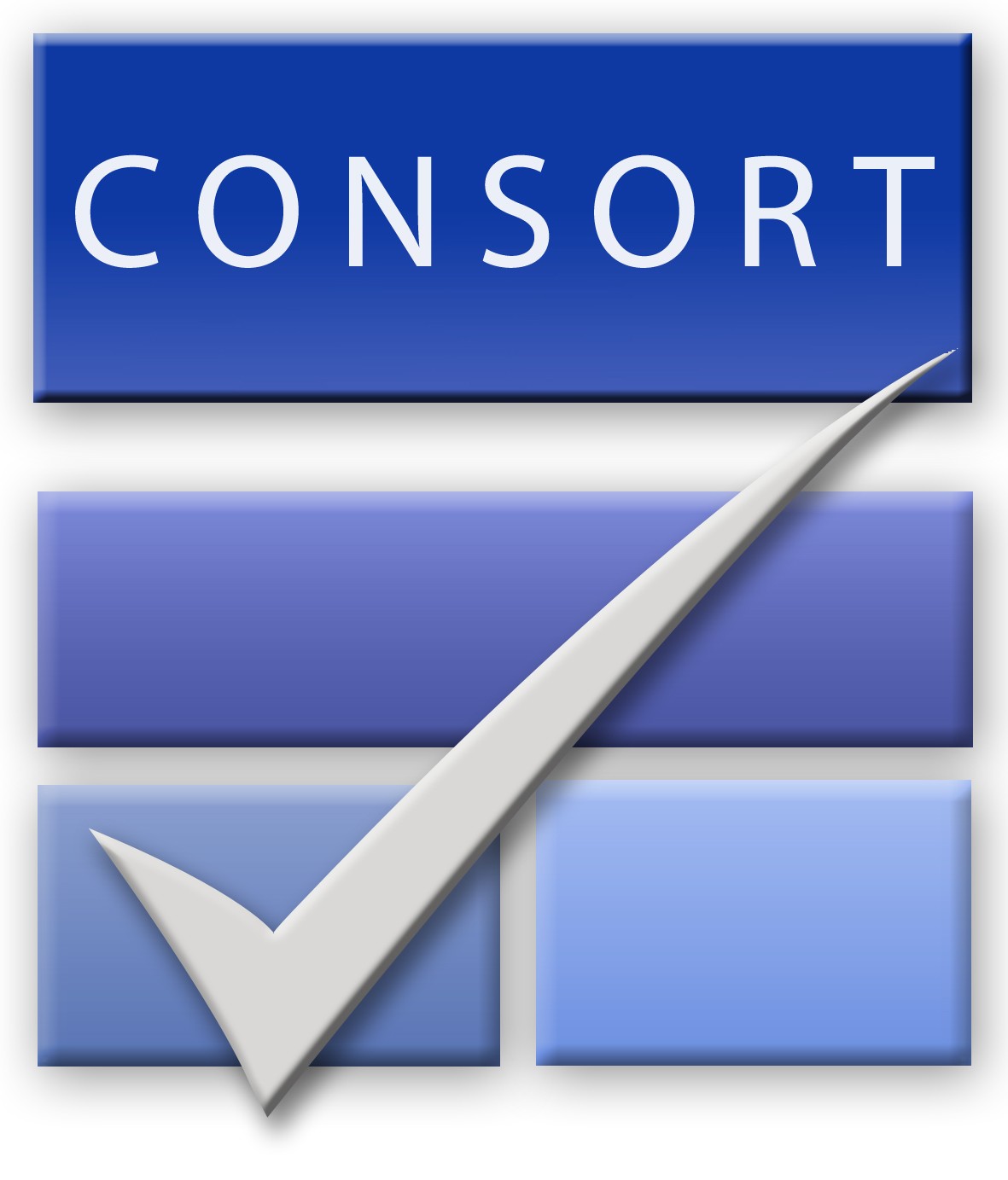
CONSORT 2010 checklist of information to include when reporting a randomised trial*

| Section/Topic | Item No | Checklist Item | Reported on page No |
| --- | --- | --- | --- |
| Title and abstract | | | |
|  | 1a | Identification as a randomised trial in the title | 1 |
|  | 1b | Structured summary of trial design, methods, results, and conclusions (for specific guidance see CONSORT for abstracts) | 2,3 |
| Introduction | | | |
| Background and objectives | 2a | Scientific background and explanation of rationale | 4-7 |
|  | 2b | Specific objectives or hypotheses | 7 |
| Methods | | | |
| Trial design | 3a | Description of trial design (such as parallel, factorial) including allocation ratio | 7 |
|  | 3b | Important changes to methods after trial commencement (such as eligibility criteria), with reasons | 12 |
| Participants | 4a | Eligibility criteria for participants | 8 |
|  | 4b | Settings and locations where the data were collected | 7 |
| Interventions | 5 | The interventions for each group with sufficient details to allow replication, including how and when they were actually administered | 8,9; SM 2,3,4. |
| Outcomes | 6a | Completely defined pre-specified primary and secondary outcome measures, including how and when they were assessed | 9-12 |
|  | 6b | Any changes to trial outcomes after the trial commenced, with reasons | 12 |
| Sample size | 7a | How sample size was determined | 12,13 |
|  | 7b | When applicable, explanation of any interim analyses and stopping guidelines | n/a |
| Randomisation: |  |  |  |
| Sequence  generation | 8a | Method used to generate the random allocation sequence | 8 |
|  | 8b | Type of randomisation; details of any restriction (such as blocking and block size) | 8; SM 1,2 |
| Allocation  concealment  mechanism | 9 | Mechanism used to implement the random allocation sequence (such as sequentially numbered containers), describing any steps taken to conceal the sequence until interventions were assigned | SM 2 |
| Implementation | 10 | Who generated the random allocation sequence, who enrolled participants, and who assigned participants to interventions | SM 2 |

| Blinding | 11a | If done, who was blinded after assignment to interventions (for example, participants, care providers, those assessing outcomes) and how | 8;m SM 2 |
| --- | --- | --- | --- |
|  | 11b | If relevant, description of the similarity of interventions | n/a |
| Statistical methods | 12a | Statistical methods used to compare groups for primary and secondary outcomes | 12,13 |
|  | 12b | Methods for additional analyses, such as subgroup analyses and adjusted analyses | 13; SM 5-7 |
| Results | | | |
| Participant flow (a diagram is strongly recommended) | 13a | For each group, the numbers of participants who were randomly assigned, received intended treatment, and were analysed for the primary outcome | Figure 1, Table 1;SM 1 |
|  | 13b | For each group, losses and exclusions after randomisation, together with reasons | Figure 1 |
| Recruitment | 14a | Dates defining the periods of recruitment and follow-up | 7; SM1,2 |
|  | 14b | Why the trial ended or was stopped | n/a |
| Baseline data | 15 | A table showing baseline demographic and clinical characteristics for each group | SM Table S1 |
| Numbers analysed | 16 | For each group, number of participants (denominator) included in each analysis and whether the analysis was by original assigned groups | SM 1,5,6,7 |
| Outcomes and estimation | 17a | For each primary and secondary outcome, results for each group, and the estimated effect size and its precision (such as 95% confidence interval) | 13,14,15 |
|  | 17b | For binary outcomes, presentation of both absolute and relative effect sizes is recommended | 14 |
| Ancillary analyses | 18 | Results of any other analyses performed, including subgroup analyses and adjusted analyses, distinguishing pre-specified from exploratory | n/a |
| Harms | 19 | All important harms or unintended effects in each group (for specific guidance see CONSORT for harms) | SM 4 |
| Discussion | | | |
| Limitations | 20 | Trial limitations, addressing sources of potential bias, imprecision, and, if relevant, multiplicity of analyses | 17 |
| Generalisability | 21 | Generalisability (external validity, applicability) of the trial findings | 17 |
| Interpretation | 22 | Interpretation consistent with results, balancing benefits and harms, and considering other relevant evidence | 15,16,18 |
| Other information | | |  |
| Registration | 23 | Registration number and name of trial registry | 2, 7 |
| Protocol | 24 | Where the full trial protocol can be accessed, if available | 7 |
| Funding | 25 | Sources of funding and other support (such as supply of drugs), role of funders | 19 |

*We strongly recommend reading this statement in conjunction with the CONSORT 2010 Explanation and Elaboration for important clarifications on all the items. If relevant, we also recommend reading CONSORT extensions for cluster randomised trials, non-inferiority and equivalence trials, non-pharmacological treatments, herbal interventions, and pragmatic trials. Additional extensions are forthcoming: for those and for up to date references relevant to this checklist, see [www.consort-statement.org](http://www.consort-statement.org).
